# Draft Genome Sequence of *Ignatzschineria* sp. Strain RMDPL8A, a Bacterium Isolated from Landfill Leachate

**DOI:** 10.1128/mra.00307-23

**Published:** 2023-06-05

**Authors:** Yogesh S. Nimonkar, Swapnil Kajale, Manjusha Dake, Om Prakash

**Affiliations:** a National Centre for Microbial Resource (NCMR), National Centre for Cell Science, Pune, Maharashtra, India; b Institute of Soil, Water and Environmental Sciences, Volcani Institute, Agricultural Research Organization, Rishon LeZion, Israel; c Protein Biochemistry Laboratory, Dr. D. Y. Patil Biotechnology and Bioinformatics Institute, Pune, India; d Symbiosis Centre for Climate Change and Sustainability (SCCCS), Symbiosis International (Deemed University), Lavale, Pune, India; University of Southern California

## Abstract

The genome of *Ignatzschineria* sp. strain RMDPL8A was sequenced and analyzed. This draft genome sequence was 2,175,527 bp long, with a GC content of 45.12% and 1,890 protein coding genes.

## ANNOUNCEMENT

The genus *Schineria* was first described by Tóth et al. in 2001 and later replaced with the genus *Ignatzschineria* in the order *Lysobacterales* by Tóth et al. in 2007 (1, 2). Members of the genus *Ignatzschineria* (class *Gammaproteobacteria*) are Gram-negative, aerobic, nonmotile, non-spore-forming bacteria with a regular rod shape (3). This study reports the isolation and genome sequencing of *Ignatzschineria* sp. strain RMDPL8A. The present strain was previously isolated during a landfill leachate study (4) and deposited at the National Centre for Microbial Resource (NCMR-NCCS) under the accession number MCC 5247.

The culture was revived from glycerol stock, and its purity was rechecked by restreaking it onto fresh medium plates. For genomic DNA extraction, the culture was grown in tryptic soya broth (TSB; HiMedia; M1968-500G) and incubated at 30°C for 48 h. Cells were harvested and high-quality genomic DNA was extracted using the following method. A single bacterial colony was suspended in 500 μL of Tris EDTA (pH 8.0), followed by bead beating for 2 to 3 min. Then, proteinase K (20 mg/mL) was added, and the suspension was incubated at 55°C for 2 h with occasional shaking. A 100-μL volume of 0.5 M NaCl was added, and the suspension was incubated for 30 min at 72°C. DNA was extracted using phenol-chloroform-isoamyl alcohol (25:24:1), washed with 70% ethanol, and dissolved in 100 μL Tris-EDTA buffer (pH 8.0). An intact band of DNA was observed using ethidium bromide gel electrophoresis (5, 6).

Illumina library preparation was carried out using the Nextera DNA Flex library preparation kit (Illumina Inc., San Diego, CA, USA). The genomic DNA was sequenced on the Illumina MiSeq platform using paired-end (2 × 250-bp) technology with v2 Illumina chemistry (7). The genome quality was checked using the FastQC v0.11.9 tool (8); the raw reads were assembled using the Unicycler v0.4.8 assembler and polished using Pilon v1.23 in the PATRIC v3.6.12 online server (9). The MeDuSa Web server (10) was used for genome finishing, and the assembly quality was evaluated using the tools QUAST v5.1.0rc1 (11) and CheckM v1.2.0 (12). The NCBI Prokaryotic Genome Annotation Pipeline (PGAP) v6.3 (13) was used to annotate the genome. In all, 1,284,736 reads and 297,784,890 bases were generated, with 73× genome coverage. Default parameters were used for all software unless otherwise noted.

The total length of the genome sequence was 2,175,527 bp; sequencing yielded 3 contigs (*N*_50_, 1,848,963), with a GC content of 45.13%, 100% completeness, and 0% contamination. Among the 1,957 total genes, 1,909 coding sequences and 1,890 proteins were identified. In addition, 41 tRNAs and 2 rRNAs (one 16S rRNA and one 23S rRNA) were found. Three clustered regularly interspaced short palindromic repeat (CRISPR) arrays were found in the genome using CRISPRCasFinder (14).

### Data availability.

This whole-genome shotgun sequencing project has been deposited at DDBJ/ENA/GenBank under the accession number JAPPWA000000000. The version described in this paper is version JAPPWA010000000. The associated BioProject and Sequence Read Archive accession numbers are PRJNA907172 and SAMN31953526, respectively.
